# Wanted, a Hospital Architect

**Published:** 1913-03-08

**Authors:** 


### THE EDITOR'S LETTER-BOX.

### Wanted, a Hospital Architect.

*To the Editor of* The Hospital.

Sir,—On page 529 of The Hospital for February 15 I find an article headed "An Alternative to Clear Windows." It refers to "a block of buildings giving anything but a cheerful prospect from its windows," which "occurs in every institution, and particularly in those situated in large towns." It goes on to say "such a block is rarely found in the wards themselves," whatever that may mean, but is "usually in the staff-room block, in the nurses' or sisters' sitting-rooms." I fancy your readers must have rubbed their eyes when they read the foregoing, for the article, judging from the technical words used, would seem to have come from an architectural source. If so, most hospital workers will cry shame on the architects for ever passing plans for hospital buildings of the description indicated, and upon the mind which contemplates obscured glass in windows as a remedy for the absence of a cheerful prospect, or for an outlook on to an ugly brick boundary wall. I have had a good deal to do with sites in large towns and with some hospital sites, and I make bold to say that nowadays any architect who submitted plans for buildings containing the defects described in your article, and most certainly any architect who was content to treat an evil of the kind mentioned by obscured glass, deserves to be warned off all hospital work for all time.

The attention of the whole hospital world should be concentrated on a discussion which is now arising in connection with tuberculosis hospital and sanatorium construction. Are the buildings of the existing hospitals for consumption, like that at Mount Vernon, Hampstead, to be scrapped as too costly, and if so what kind of building is to be substituted for them? Mount Vernon Hospital as it stands is excellent in every way for its purpose. The work it has done and is doing is excellent, too. What threatens its continuance is its costly character. Here is an object-lesson for all hospital managers, and especially for hospital committees who have reconstructions or new buildings to decide upon and provide for. In the United States this question of buildings for tuberculosis hospitals is being handled with energetic and economic directness which seems calculated to create a revolution in regard to expensive buildings.* At any rate, evidence accumulates that the present need of the day in regard to hospital construction is the advent of an architect and a hospital expert who will devote themselves to the production of up-to-date plans and estimates and an adequate system of hospital construction which will reduce the cost of these structures by at least 50 to 75 per cent. of the amounts now prevailing in architectural circles. I am aware that local by-laws, building regulations, and other routine matters will probably be urged as making this proposal impracticable. My answer must be, no one who has studied the existing by-laws, and who realises the importance of the maximum of sunlight and the best of air for buildings containing patients needing up-to-date treatment, can doubt for a moment that hospital sites for town residents must be selected outside city boundaries in the near future, in accordance with the plan fully set forth in the *Encyclopædia Britannica*, eleventh edition, vol. 13, pages 791 to 800 inclusive. The old system of costly buildings, obscured-glass windows, deficient sunlight, inadequate air supply, and enormous cost is condemned, and unless the architectural profession can produce a man or men to grasp this fact and show the way to better things, science, through the universities, will take up the question, and this branch of work may be taken out of the hands of the architectural profession altogether.—I am, Sir, yours obediently, "One Who Knows."

* See Sanatoria Buildings at £50 per Bed, page 608.

